# Impact of gadolinium‐ethoxybenzyl‐diethylenetriamine pentaacetic acid‐enhanced magnetic resonance imaging on the prognosis of hepatocellular carcinoma after surgery

**DOI:** 10.1002/jgh3.12444

**Published:** 2020-10-30

**Authors:** Shingo Shimada, Toshiya Kamiyama, Tatsuhiko Kakisaka, Tatsuya Orimo, Akihisa Nagatsu, Yoh Asahi, Yuzuru Sakamoto, Daisuke Abo, Hirofumi Kamachi, Akinobu Taketomi

**Affiliations:** ^1^ Department of Gastroenterological Surgery I Hokkaido University Graduate School of Medicine Sapporo Japan; ^2^ Department of Diagnostic Imaging Hokkaido University Graduate School of Medicine Sapporo Japan

**Keywords:** hepatocellular carcinoma, magnetic resonance imaging, portal venous invasion, Primovist

## Abstract

**Background and Aim:**

Gadolinium‐ethoxybenzyl‐diethylenetriamine pentaacetic acid (Gd‐EOB‐DTPA)‐enhanced magnetic resonance imaging (EOB‐MRI) has been recognized as a useful imaging technique to distinguish the biological behavior of hepatocellular carcinoma (HCC).

**Methods:**

We analyzed 217 hepatectomy recipients with HCCs measuring 10 cm or less. We divided the patients into a decreased intensity (DI) group (*n* = 189, 87%) and an increased or neutral intensity (INI) group (*n* = 28, 13%) according to the ratio of tumor intensity to liver intensity during the hepatobiliary phase (HBP). According to the ratio of the maximum tumor diameter (including peritumoral hypointensity) between HBP images and precontrast T1‐weighted images (RHBPP), we divided the patients as follows: The group whose RHBPP was ≥1.036 was the high RHBPP group (*n* = 60, 28%), and the group whose RHBPP was <1.036 was the low RHBPP group (*n* = 157, 72%). We investigated the prognoses and clinicopathological characteristics of these patients.

**Results:**

DI *versus* INI was not a prognostic factor for either survival or recurrence; however, a high RHBPP was an independent predictor of unfavorable survival and recurrence in patients. In addition, the INI group showed significantly lower α‐fetoprotein (AFP) levels and higher rates of well‐differentiated HCC and ICGR15 ≥15% than the DI group. The high RHBPP group showed significantly higher rates of vascular invasion and poorly differentiated HCC than the low RHBPP group.

**Conclusions:**

A high RHBPP by EOB‐MRI is a preoperative predictor of vascular invasion and an unfavorable prognostic factor for survival and recurrence. These patients might be considered for highly curative operations such as anatomical liver resection.

## Graphical abstract

We analyzed 217 hepatectomy recipients with hepatocellular carcinomas measuring 10 cm or less in diameter based on the findings of Gd‐EOB‐DTPA‐enhanced magnetic resonance imaging (EOB‐MRI). The characteristics of EOB‐MRI are useful as a predictor for vascular invasion and its outcome.

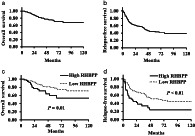

## Introduction

Liver cancer is the seventh most frequent type of cancer, with an estimated 841 080 cases per year, and it is the second leading cause of cancer‐related death, causing approximately 781 631 deaths per year.^1^ Hepatocellular carcinoma (HCC) has a poor prognosis and accounts for 70–85% of primary liver cancers.^2^ Curative hepatectomy for HCC is a useful method for achieving long‐term survival.^3^ Before planning to perform hepatectomy for HCC, clinicians generally perform imaging studies such as ultrasonography (US), contrast‐enhanced computed tomography (CT), and contrast‐enhanced magnetic resonance imaging (MRI).

Recently, gadolinium‐ethoxybenzyl‐diethylenetriamine pentaacetic acid (Gd‐EOB‐DTPA)‐enhanced MRI (EOB‐MRI) has become a common tool for assessing hepatectomy candidates. Gd‐EOB‐DTPA is a liver‐specific paramagnetic gadolinium‐based contrast agent used exclusively in magnetic resonance liver imaging. This agent not only pervades the vascular and extravascular space as effectively as general gadolinium‐based contrast agents but also progressively enters the hepatocytes and bile ducts during the hepatobiliary phase (HBP) (20 min after Gd‐EOB‐DTPA injection). Thus, EOB‐MRI is recognized as a useful modality to obtain additional information about the characterization and detection of liver tumors.^4^ Regarding EOB‐MRI, previous studies have reported a sensitivity of 92–95% and a specificity of 95–98% for the detection of HCC.^5^ In general, HCCs show lower intensity than the liver parenchyma during the HBP.^6^ However, some HCCs show the same or higher intensity than the liver parenchyma during the HBP.^7^, ^8^ Previous reports have shown differences in the biological behavior of HCCs.^9^, ^10^ Moreover, it has been reported that peritumoral hypointensity during the HBP correlates with microvascular invasion.^11^ However, there are relatively few reports regarding the correlation between these findings and the prognoses or clinicopathological characteristics of hepatectomy recipients.

In the current study, we investigated the impact of preoperative EOB‐MRI findings on prognosis and the correlation between these findings and the clinicopathological characteristics of HCC patients.

## Methods

### *Patients*

Between January 2008 and December 2018, we performed primary liver resection for 282 consecutive patients with single HCCs that measured 10 cm or less in diameter at the Gastroenterological Surgery I Unit of Hokkaido University Hospital in Sapporo, Japan. We selected only patients with single HCCs because we intended to focus on only one tumor for evaluation. We excluded 65 patients who did not undergo an EOB‐MRI evaluation, leaving 217 patients for analysis.

### *Technique and analysis*

EOB‐MRI was performed 8 days (median, range 1–92) before hepatectomy. MRI was performed using a 1.5‐T system (Achieva A‐series, Phillips Medical Systems, Best, The Netherlands) with a 32‐channel cardiac phased‐array coil. Our routine protocol for liver MRI sequences was as follows: breath‐hold T1‐weighted dual gradient‐recalled echo (GRE) in‐phase and opposed‐phase imaging, a respiratory‐triggered T2‐weighted fast spin‐echo sequence, a half‐Fourier acquisition single‐shot turbo spin‐echo (HASTE) sequence, and diffusion‐weighted imaging (DWI; b values of 0 and 1000 s/mm^2^). For dynamic imaging, 0.1 mL/kg of gadoxetic acid (Primovist, Bayer Healthcare) was administered intravenously at a rate of 1.0 mL/s, followed by a 40‐mL saline flush. Pre‐ and postcontrast images using a T1‐weighted, fat‐suppressed three‐dimensional (3D) GRE sequence were obtained. The arterial phase began when the contrast medium reached the distal thoracic aorta (approximately 30 s after injection of the contrast medium), as observed by real‐time MRI fluoroscopic monitoring, and the portal venous phase, delayed phase, and HBP were defined as approximately 50–60 s, 3 min, and 20 min after injection of the contrast medium, respectively.

The signal intensity (SI) of each region of interest (ROI) was measured from HBP images on a DICOM viewer. The SI of the tumor was measured once in a circular area (1–2 cm^2^) at the level of the maximum tumor diameter. The SI of the nontumorous liver parenchyma was measured in three different circular areas (each measuring 1–2 cm^2^) and placed at the same level (maximum tumor diameter) to avoid vascular structures, artifacts, and focal liver lesions. The SI value of the nontumorous liver parenchyma was calculated as the average of the three sample areas. We calculated the ratio of the SI value of the tumor to that of the nontumorous liver parenchyma and defined it as the tumor–liver parenchyma index (TLPI).

**Figure 1:**
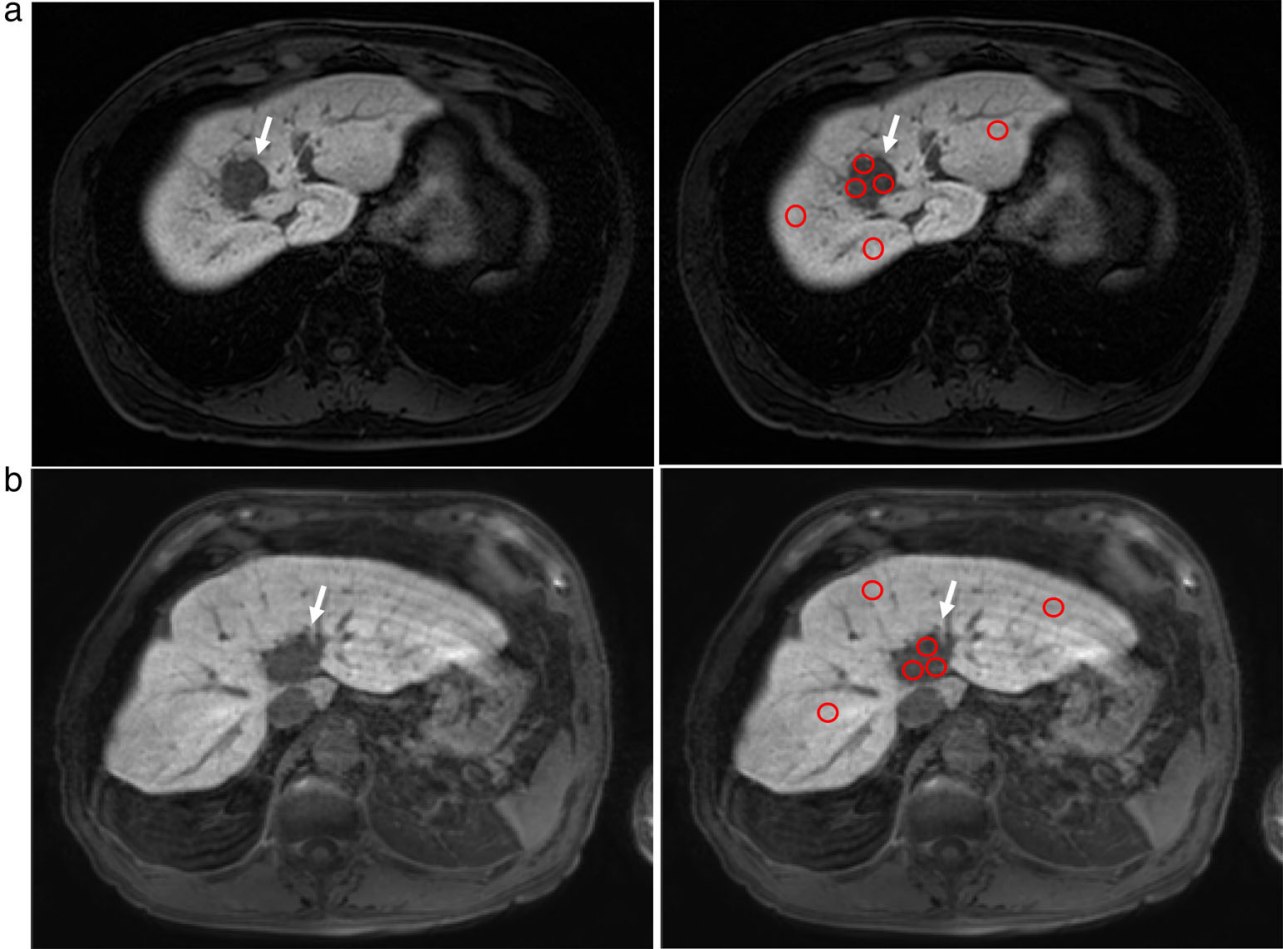
(a) A male in his 60s with hepatocellular carcinoma (HCC). Signal intensity (SI) is decreased in the tumor (white arrow) compared with that in the liver parenchyma, as shown on hepatobiliary phase (HBP) imaging. The tumor–liver parenchyma index (TLPI) was 0.3901. The SI of each region of interest (ROI) is shown by red circles. (b) A male in his 80s with HCC. The SI was decreased in the tumor (white arrow) compared with that in the liver parenchyma, as shown on HBP imaging. The TLPI was 0.3945. The SI of each ROI is shown by red circles.

**Figure 2:**
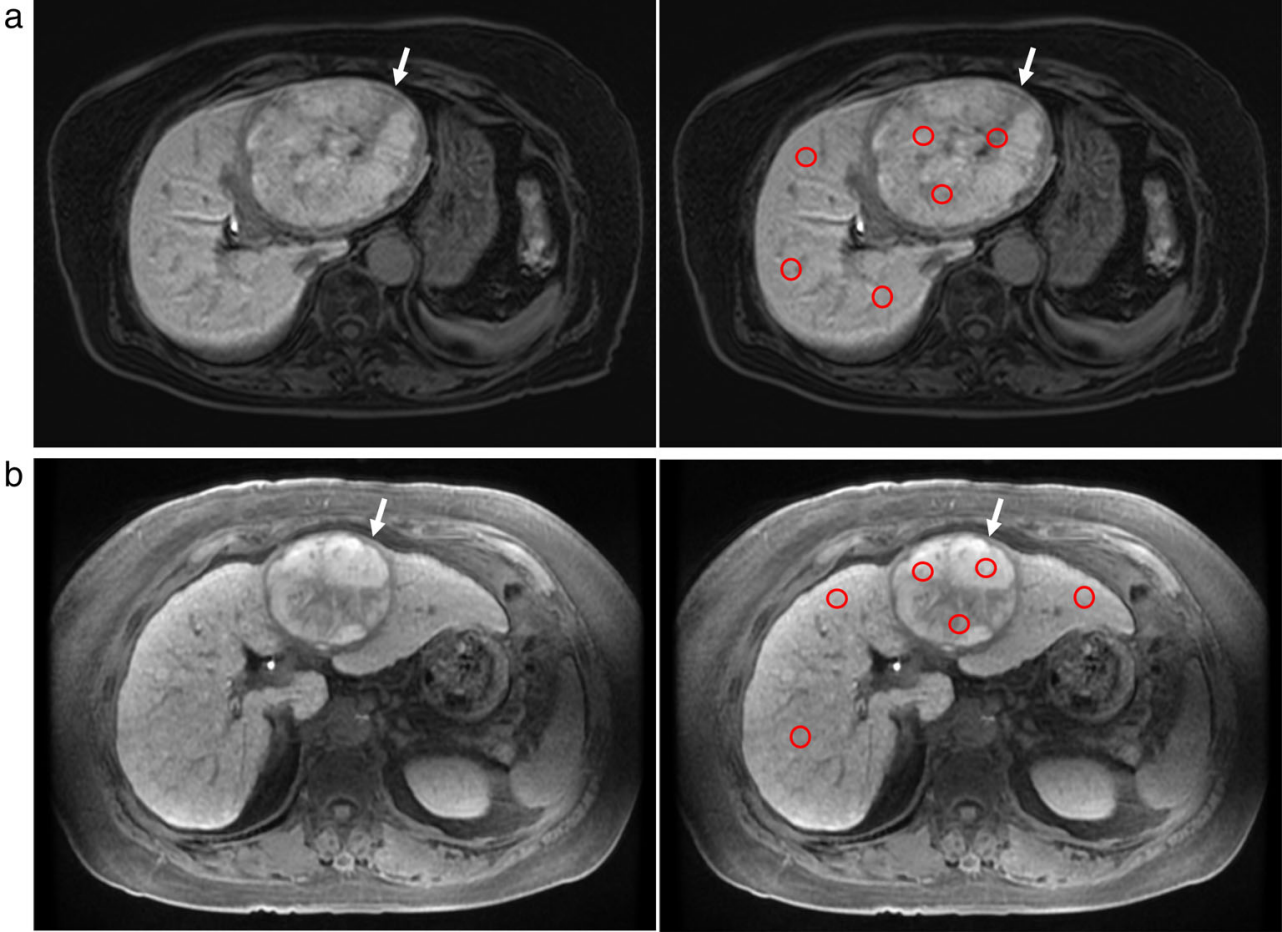
(a) A male in his 80s with hepatocellular carcinoma (HCC). The signal intensity (SI) was increased or neutral in the tumor (white arrow) compared with that in the liver parenchyma, as shown on hepatobiliary phase (HBP) imaging. The tumor–liver parenchyma index (TLPI) was 1.1610. The SI of each region of interest (ROI) is shown by red circles. (b) A female in her 60s with HCC. The SI was increased or neutral in the tumor (white arrow) compared with that in the liver parenchyma, as shown on HBP imaging. The TLPI was 1.0755. The SI of each ROI is shown by red circles.

First, we defined the patients whose TLPI was <0.9, that is, the SI value of the tumor was decreased compared with that of the nontumorous liver parenchyma, as having decreased tumor SI (decreased intensity (DI) group; *n* = 189, 87%) (Fig. 1); the patients whose TLPI was ≥0.9, that is, those whose TLPI was increased or neutral, were defined as having increased or neutral tumor SI (increased or neutral intensity (INI) group; *n* = 28, 13%) (Fig. 2).

**Figure 3:**
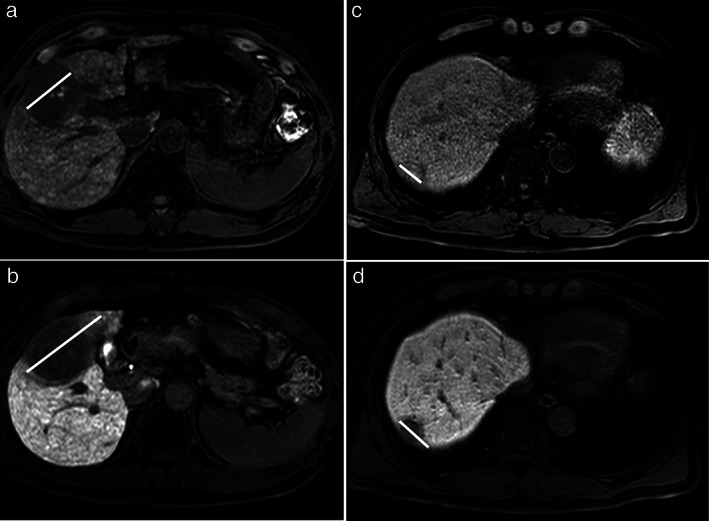
(a) A male in his 60s with hepatocellular carcinoma (HCC). Precontrast enhanced T1‐weighted image showing an inhomogeneous tumor in hepatic segment V. The maximum tumor diameter was 55 mm. (b) Hepatobiliary phase (HBP) images (same patient as described in a) showing low SI of the tumor. In addition, a portion showing slight hypointensity around the tumor (peritumoral hypointensity) is noted. The diameter was measured so that the maximum tumor diameter with peritumoral hypointensity was included (64 mm). The ratio of the maximum tumor diameter including peritumoral hypointensity between HBP images and precontrast T1‐weighted images (RHBPP) was 1.163. (c) A male in his 50s with HCC. Precontrast enhanced T1‐weighted image showing an inhomogeneous tumor in hepatic segment VII. The maximum tumor diameter was 25 mm. (d) HBP images (same patient as described in c) showing low SI of the tumor. In addition, a portion showing peritumoral hypointensity is noted. The diameter was measured so that the maximum tumor diameter with peritumoral hypointensity was included (37 mm). The RHBPP was 1.480.

**Figure 4:**
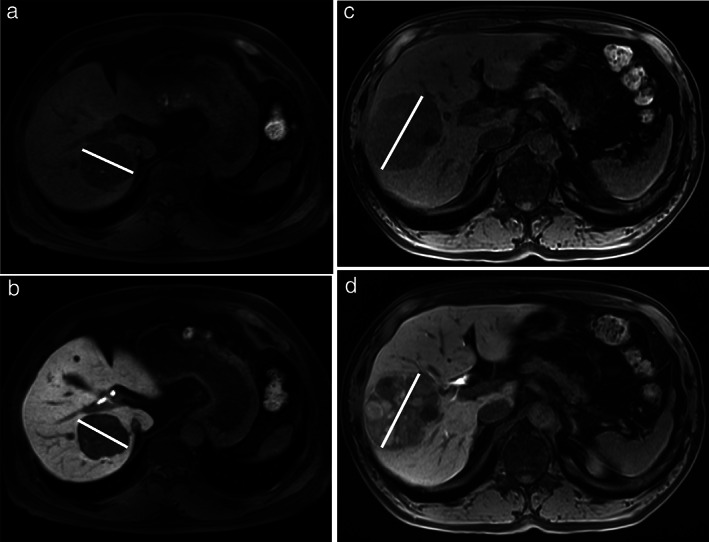
(a) A male in his 60s with hepatocellular carcinoma (HCC). Precontrast enhanced T1‐weighted image showing an inhomogeneous tumor in hepatic segments VI and VII. The maximum tumor diameter was 53 mm. (b) Hepatobiliary phase (HBP) images (same patient as described in Fig. 4a) showing low signal intensity (SI) in the tumor without peritumoral hypointensity. The maximum tumor diameter was 53 mm. The RHBPP was 1.000. (c) A male in his 70s with HCC. Precontrast enhanced T1‐weighted image showing an inhomogeneous tumor in hepatic segments VII and VIII. The maximum tumor diameter was 77 mm. (d) HBP images (same patient as described in Fig. 4c) showing low SI in the tumor without peritumoral hypointensity. The maximum tumor diameter was 74 mm. The RHBPP was 0.9610.

Second, we calculated the ratio of the maximum tumor diameter, including the peritumoral hypointensity (the portion showing hypointensity around the tumor), on HBP images to the corresponding diameter measured on precontrast T1‐weighted images and defined this ratio as the RHBPP. We defined the group with an RHBPP ≥1.036 as the high RHBPP group (*n* = 60, 28%) (Fig. 3) and those with an RHBPP <1.036 as the low RHBPP group (*n* = 157, 72%) (Fig. 4) using a receiver operating characteristic (ROC) curve for the presence of portal venous invasion (PVI).

We compared clinicopathological characteristics between these groups. In addition, we investigated the preoperative prognostic factors for survival and recurrence, including the RHBPP and DI, on EOB‐MRI.

The indications for hepatectomy were usually determined based on the patients' liver function reserve, that is, the results of the indocyanine green retention test at 15 min (ICGR15).^12^ Essentially, the patients in whom the ICGR15 was lower than 25% underwent anatomical resection.

Nonanatomical partial hepatectomy with complete tumor resection was performed in the other patients. In all hepatectomies performed, the resection surfaces were found to be histologically and macroscopically free of HCC. We conducted follow‐up analyses every 3 months after hepatectomy; these analyses included physical, serological (serum α‐fetoprotein [AFP], serum protein induced by vitamin K absence‐II [PIVKA‐II], and liver function test), and radiological (CT along with US or MRI) examinations. Recurrence was diagnosed from the results of radiological examinations and elevations in the serum levels of AFP and/or PIVKA‐II. Extrahepatic metastasis was diagnosed by chest and abdominal CT, contrast‐enhanced brain MRI, and bone scintigrams. The median follow‐up period was 32 months (range, 1.0–128 months). The correlation coefficient between the maximum tumor size on precontrast T1‐weighted images and that of the liver resection specimen was 0.9510.

This study was approved by the Hokkaido University Hospital Voluntary Clinical Study Committee (approval 019‐0195) and performed in accordance with the guidelines set forth by the Declaration of Helsinki.

### *Statistical analysis*

The clinicopathological characteristics of the high RHBPP group were compared with those of the low RHBPP group, and those of the INI group were compared with those of the DI group. Univariate comparisons between two groups were performed using the Mann–Whitney U test for continuous variables and the chi‐squared test for noncontinuous variables. Overall survival (OS) and relapse‐free survival (RFS) were determined by the Kaplan–Meier method and analyzed with the log‐rank test or Cox proportional hazards model. Pearson's correlation coefficient was used to analyze the correlation between the maximum tumor size on precontrast T1‐weighted images and those of the liver resection specimen. Statistical analyses were performed using JMP Pro 14.0.0 for Windows (SAS Institute, Cary, NC, USA). Significance was defined as a *P*‐value <0.05.

## Results

### *Clinicopathological characteristics and prognoses in this cohort*

**Figure 5:**
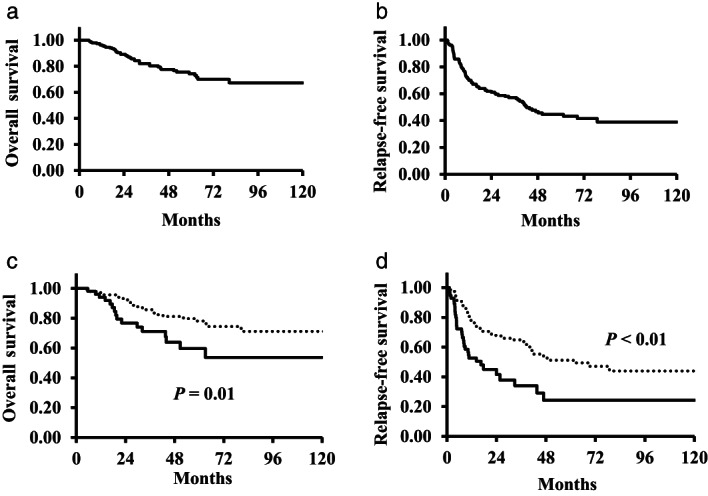
(a) Overall survival curves of this cohort. (b) Relapse‐free survival curves of this cohort. (c) Overall survival curves of patients with a high RHBPP (ratio of the maximum tumor diameter including peritumoral hypointensity between HBP images and precontrast T1‐weighted images) and those with a low RHBPP. (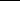), High RHBPP; (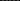), low RHBPP. (d) Relapse‐free survival curves of patients with a high RHBPP and those with a low RHBPP. (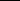), High RHBPP; (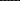), low RHBPP

Table 1 shows the clinicopathological characteristics in this cohort. The 5‐year OS rate was 73.5%, the 5‐year RFS rate was 44.5%, and the median relapse‐free time was 42 months (Fig. 5a,b).

**Table 1:** Clinicopathological characteristics of patients in this study

Characteristic	All patients (*n* = 217)
Epidemiology	
Age	
<60	35 (16%)
≥60	182 (84%)
Gender	
Male	177 (82%)
Female	40 (18%)
HBs‐Ag	
Positive	59 (27%)
Negative	158 (73%)
HCV‐Ab	
Positive	54 (25%)
Negative	163 (75%)
NBNC	
Yes	106 (49%)
No	111 (51%)
Biochemical factors	
Platelets	
<80,000/mm^3^	12 (6%)
≥80,000/mm^3^	205 (94%)
Albumin	
<3.5 g/dL	16 (7%)
≥3.5 g/dL	201 (93%)
Total bilirubin	
≥1.0 mg/dL	42 (19%)
<1.0 mg/dL	175 (81%)
PT	
<80%	21 (10%)
≥80%	196 (90%)
ChE	
<250 IU/L	78 (36%)
≥250 IU/L	139 (64%)
ICGR15	
≥15%	94 (43%)
<15%	123 (57%)
AFP	
≥20 ng/mL	71 (33%)
<20 ng/mL	146 (67%)
PIVKA‐II	
≥100 mAU/mL	109 (50%)
<100 mAU/mL	108 (50%)
Tumor factors	
Tumor size	
≥5 cm	75 (35%)
<5 cm	142 (65%)
Macroscopic type	
Simple nodular	109 (50%)
Others	108 (50%)
Histological factors	
Growth type	
Expansive growth	211 (97%)
Invasive growth	6 (3%)
Tumor capsule	
fc(+)	153 (71%)
fc(−)	64 (29%)
Separation form	
sf(+)	144 (66%)
sf(−)	73 (34%)
Serosa infiltration	
s0	199 (92%)
s1–3	18 (8%)
Differentiation	
poor	77 (35%)
Others	140 (65%)
PVI	
Yes	42 (19%)
No	175 (81%)
HVI	
Yes	19 (9%)
No	198 (91%)
Fibrosis	
f3/4	78 (36%)
f0–2	139 (64%)
EOB‐MRI	
RHBPP	
≥1.036	60 (28%)
<1.036	157 (72%)
DI or INI	
DI	189 (87%)
INI	28 (13%)

AFP, alpha fetoprotein; ChE, cholinesterase; DI, decreased tumor intensity compared with liver intensity; f3, bridging fibrosis; f4, cirrhosis; HBs‐Ag, HBs‐antigen; HCV‐Ab, HCV antibody; HVI, hepatic venous invasion; ICGR15, indocyanine green retention rate at 15 min; INI, increased or neutral tumor intensity compared with liver intensity; NBNC, patients without HBV and HCV; PT, prothrombin time; PIVKA‐II, protein induced by vitamin K absence‐II; PVI, portal venous invasion; RHBPP, ratio of the maximum tumor diameter including peritumoral hypointensity (the portion showing hypointensity around the tumor) measured on HBP image to the maximum tumor diameter measured on precontrast T1‐weighted images.

### *Differences in clinicopathological characteristics between the* *DI* *and* *INI* *groups*

The median TLPI was 0.5099 (0.2175–2.4945).

The INI group showed significantly lower AFP levels, a higher rate of well‐differentiated HCC, and a higher rate of ICGR15 ≥15% than the DI group. There were no significant differences in the other factors (Table 2).

**Table 2:** Clinicopathological characteristics of patients in the decreased tumor intensity compared with liver intensity (DI) and increased or neutral tumor intensity compared with liver intensity (INI) groups

Characteristic	DI (*n* = 189)	INI (*n* = 28)	*P*
Epidemiology			
Age			
<60	33 (17%)	2 (7%)	0.16
≥60	156 (83%)	26 (93%)	
Gender			
Male	157 (83%)	20 (71%)	0.13
Female	32 (17%)	8 (29%)	
HBsAg			
Positive	54 (29%)	5 (18%)	0.23
Negative	135 (71%)	23 (82%)	
HCV‐Ab			
Positive	47 (25%)	7 (25%)	0.98
Negative	142 (75%)	21 (75%)	
NBNC			
Yes	88 (47%)	18 (64%)	0.07
No	101 (53%)	10 (36%)	
Biochemical factors			
Platelets			
<80 000/mm^3^	11 (6%)	1 (4%)	0.62
≥80 000/mm^3^	178 (94%)	27 (96%)	
Albumin			
<3.5 g/dL	12 (6%)	4 (14%)	0.13
≥3.5 g/dL	177 (94%)	24 (86%)	
Total bilirubin			
≥1.0 mg/dL	37 (20%)	5 (18%)	0.82
<1.0 mg/dL	152 (80%)	23 (82%)	
PT			
<80%	17 (9%)	4 (14%)	0.37
≥80%	172 (91%)	24 (86%)	
ChE			
<250 IU/L	66 (35%)	12 (43%)	0.41
≥250 IU/L	123 (65%)	16 (57%)	
ICGR15			
≥15%	76 (40%)	18 (64%)	0.01
<15%	113 (60%)	10 (36%)	
AFP			
≥20 ng/mL	69 (37%)	2 (7%)	<0.01
<20 ng/mL	120 (63%)	26 (93%)	
PIVKA‐II			
≥100 mAU/mL	94 (50%)	15 (54%)	0.70
<100 mAU/mL	95 (50%)	13 (46%)	
Tumor factors			
Tumor size			
≥5 cm	65 (34%)	10 (36%)	0.68
<5 cm	124 (66%)	18 (64%)	
Macroscopic type			
Simple nodular	92 (49%)	17 (61%)	0.23
Others	97 (51%)	11 (39%)	
Histological factors			
Growth type			
Expansive growth	183 (97%)	28 (100%)	0.33
Invasive growth	6 (3%)	0 (0%)	
Tumor capsule			
fc(+)	129 (68%)	23 (82%)	0.13
fc(−)	60 (32%)	5 (18%)	
Separation form			
sf(+)	127 (67%)	17 (61%)	0.49
sf(−)	62 (33%)	11 (39%)	
Serosa infiltration			
s0	171 (90%)	28 (100%)	0.08
s1–3	18 (10%)	0 (0%)	
Differentiation			
Well	32 (17%)	14 (40%)	<0.01
Others	157 (83%)	14 (50%)	
PVI			
Yes	38 (20%)	4 (14%)	0.46
No	151 (80%)	24 (86%)	
HVI			
Yes	19 (10%)	0 (0%)	0.07
No	170 (90%)	28 (100%)	
Fibrosis			
f3/4	64 (34%)	14 (50%)	0.09
f0–2	125 (65%)	14 (50%)	

AFP, alpha fetoprotein; ChE, cholinesterase; f3, bridging fibrosis; f4, cirrhosis; HBsAg, HBs‐antigen; HCV‐Ab, HCV antibody; HVI, hepatic venous invasion; ICGR15, indocyanine green retention rate at 15 min; NBNC, patients without HBV and HCV; PIVKA‐II, protein induced by vitamin K absence‐II; PT, prothrombin time; PVI, portal venous invasion.

### *Differences in clinicopathological characteristics between the high* *RHBPP* *and low* *RHBPP* *groups*

The high RHBPP group showed significantly higher rates of PVI, hepatic venous invasion (HVI), serosa infiltration, and poorly differentiated HCC than the low RHBPP group. There were no significant differences in other factors, such as liver function (Table S1, Supporting information).

### *Prognostic factors for* *OS* *and* *RFS, including the* *RHBPP* *and* *DI*

Table S2 shows the preoperative prognostic factors for survival and recurrence.

The univariate analysis revealed that OS was significantly related to the following factors: albumin <3.5 g/dL, prothrombin time (PT) <80%, cholinesterase (ChE) <250 IU/L, ICGR15 ≥15%, PIVKA‐II ≥100 mAU/mL, and RHBPP ≥1.036. These analyses also indicated that RFS was significantly related to albumin <3.5 g/dL, ChE <250 IU/L, ICGR15 ≥15%, AFP ≥20 ng/mL, PIVKA‐II ≥100 mAU/mL, RHBPP ≥1.036, and tumor size ≥5 cm, except in cases of the simple nodular type. A high RHBPP was an unfavorable prognostic factor for both survival and recurrence, with 5‐year OS and RFS rates of 60% (survival) and 24% (recurrence) *versus* 78% (survival) and 51% (recurrence) (*P* = 0.01, <0.01), respectively (Fig. 5c,d). However, DI was not a prognostic factor for either survival or recurrence, with 5‐year OS and RFS rates of 73% (survival) and 25% (recurrence) *versus* 85% (survival) and 47% (recurrence), respectively (data not shown).

The multivariate analysis indicated that ChE <250 IU/L, PIVKA‐II ≥100 mAU/mL, and RHBPP ≥1.036 were independent unfavorable prognostic factors for OS and that ChE <250 IU/L, PIVKA‐II ≥100 mAU/mL, RHBPP ≥1.036, and tumor size ≥5 cm were independent unfavorable prognostic factors for RFS.

## Discussion

The INI group showed significantly lower AFP levels, a higher rate of well‐differentiated HCC, and a higher ICGR15 than the DI group. The high RHBPP group showed significantly higher rates of PVI, HVI, and poorly differentiated HCC than the low RHBPP group. The results of our univariate and multivariate analyses indicate that DI *versus* INI is not a prognostic factor for either survival or recurrence; however, a high RHBPP is an independent unfavorable prognostic factor for survival and recurrence in patients with HCC. Therefore, DI and the RHBPP may be predictive factors for the malignancy of HCC.

Gd‐EOB‐DTPA is a contrast agent transported into hepatocytes by organic anion transporting polypeptide 8 (OATP8, synonymous with OATP1B3) during the HBP.^8^, ^13^ Therefore, many liver tumors show hypointensity compared with the liver parenchyma during this phase. In general, HCCs follow this pattern, that is, hypointensity compared with the liver parenchyma. However, 6–20% of HCCs have the same or higher intensity than the liver parenchyma.^9^, ^10^, ^13^, ^14^, ^15^ The rate in our study was 13%, which is consistent with previous reports.

Previous reports have shown that the overexpression of OATP8 in HCC cells is related to isointensity or hyperintensity during the HBP.^7^, ^8^, ^9^ Miura *et al*. reported that several genes associated with normal hepatic metabolism, such as alcohol dehydrogenase (ALDH)1B, the cytochrome P450 family (CYP), and solute carrier organic anion transporter family member 1B3 (SLCO1B3; synonymous with OATP8), are upregulated in HCCs showing hyperintensity during the HBP compared with HCCs showing hypointensity.^9^ SLCO1B3 is a transporter found in normal hepatocytes and is involved in the uptake of Gd‐EOB‐DTPA.^8^, ^16^ Moreover, Kitao *et al*. reported that the coactivation of β‐catenin and hepatocyte nuclear factor 4α (HNF4α) is an important regulator of OATP8 expression in HCC, and these patients showed a significantly higher differentiation grade than other patients.^10^ HNF4α expression decreases in accordance with the progression of HCC.^17^ Yamashita *et al*. reported that the knockdown of HNF4α in HCC cells showing Gd‐EOB‐DTPA uptake resulted in the increased expression of AFP.^14^ Chen *et al*. reported that the SI value of tumors during the HBP in patients with HCC with a Ki67 index >50% was significantly lower than that in patients with a Ki67 index ≤50%.^18^ In this study, the INI group showed significantly lower AFP levels and higher rates of patients with well‐differentiated HCC than the DI group. These results were consistent with those from previous reports. Some reports claimed that HCCs with iso‐ or hyperintensity during the HBP had significantly more favorable prognoses than HCCs with hypointensity.^14^, ^15^ Ariizumi *et al*. reported that hyperintense HCC was associated with better long‐term survival after curative hepatectomy than hypointense HCC.^15^ Their report also showed that the 5‐year OS and RFS rates were 100 and 56% *versus* 71 and 38%, respectively.^15^ However, our study did not show a significant difference in either survival or recurrence. The reason for this discrepancy may be influenced by the patient inclusion criteria, which were limited to a single tumor in this study as opposed to a single or multiple tumors in the study by Ariizumi *et al*.^15^ The presence of multiple tumors is itself known to be an unfavorable factor.^19^

In addition, the INI group showed a significantly higher rate of patients with an ICGR15 ≥15% than the DI group in this study. This could be because the EOB uptake of the tumor in the HBP was not dependent on liver function; however, that of the liver parenchyma was related to liver function.^20^ Therefore, the difference between the intensity of EOB uptake between the tumor and liver parenchyma during the HBP would tend to be small in patients with impaired liver function. The TLPI of these patients tends to be elevated.

The precise mechanisms of peritumoral hypointensity during the HBP are still elusive, but previous studies have proposed the following hypothesis.^11^, ^21^, ^22^ First, tumor invasion into portal branches alters the hemodynamics and perfusion of the peritumoral liver parenchyma. Second, these changes decrease the expression of OATPs or the transport protein known as multidrug resistance‐associated protein (MRP) in hepatocytes.^13^, ^21^ Therefore, the uptake of Gd‐EOB‐DTPA might be decreased due to hepatocyte dysfunction. Several reports have shown that peritumoral hypointensity during the HBP is associated with microvascular invasion.^11^, ^21^, ^22^, ^23^, ^24^, ^25^ Kim *et al*. reported that the specificity and positive predictive value were 93.2 and 88.5%, respectively.^21^

Moreover, Zhang *et al*. also reported that peritumoral hypointensity during the HBP and rim enhancement during the arterial phase were independent risk factors for microvascular invasion.^25^ They claimed that two predictors in combination identified 32.79% of HCCs with microvascular invasion, with a specificity of 95.15%, and the sensitivity and specificity of only peritumoral hypointensity were 49.18 and 89.32%, respectively.

In this study, the high RHBPP group showed significantly higher rates of vascular invasion than the low RHBPP group, which is consistent with previous studies. Although Shin *et al*. reported that the 3‐year RFS rates of patients with tumors with and without decreased peritumoral uptake were 30.8 and 63.5%, respectively (*P* < 0.01),^26^ and Ahn *et al*. reported that peritumoral hypointensity on the HBP significantly predicted early recurrence, microvascular invasion, and a high tumor grade,^24^ few reports have investigated peritumoral hypointensity associated with prognoses after hepatectomy. Our study showed that a high RHBPP was an unfavorable prognostic factor for both survival and recurrence after hepatectomy. The RHBPP is convenient and useful as a prognostic indicator. Our previous study reported that anatomical liver resection was a significantly favorable factor for both the OS and RFS of HCC patients who had a single HCC under 5 cm with microscopic PVI.^27^ Therefore, patients with a high RHBPP might be considered for anatomical liver resection because the high RHBPP group showed significantly higher rates of PVI and HVI than the low RHBPP group.

Our study has several limitations. First, some relevant genes or proteins, such as transporters, were not evaluated. Second, any tumor heterogeneity might not be reflected because the measurement of the SI of the tumor was performed at only one level using axial imaging. Third, selection bias might exist because we excluded 65 hepatectomy recipients who did not undergo EOB‐MRI. Therefore, further detailed investigations are needed.

In conclusion, an area of peritumoral hypointensity area on HBP imaging that is larger than the size of the tumor on precontrast T1‐weighted imaging is a preoperative predictor of vascular invasion and a prognostic factor for survival and recurrence. These patients might be considered for highly curative operations such as anatomical liver resection.

## Supporting information


**Table S1.** Clinicopathological characteristics of patients in the high RHBPP and low RHBPP groups.

**Table S2.** Prognostic factors for survival and recurrence.

## References

[1] Bray F , Ferlay J , Soerjomataram I , Siegel RL , Torre LA , Jemal A . Global cancer statistics 2018: GLOBOCAN estimates of incidence and mortality worldwide for 36 cancers in 185 countries. CA Cancer J. Clin. 2018; 68: 394–424. 30207593 10.3322/caac.21492

[2] Ahmed F , Perz JF , Kwong S , Jamison PM , Friedman C , Bell BP . National trends and disparities in the incidence of hepatocellular carcinoma,1998‐2003. Prev. Chronic Dis. 2008; 5: A74. 18558024 PMC2483571

[3] Shindoh J , Makuuchi M , Matsuyama Y *et al* Complete removal of the tumor‐bearing portal territory decreases local tumor recurrence and improves disease‐specific survival of patients with hepatocellular carcinoma. J. Hepatol. 2016; 64: 594–600. 26505120 10.1016/j.jhep.2015.10.015

[4] Huppertz A , Balzer T , Blakeborough A *et al* Improved detection of focal liver lesions at MR imaging: multicenter comparison of gadoxetic acid‐enhanced MR images with intraoperative findings. Radiology. 2004; 230: 266–75. 14695400 10.1148/radiol.2301020269

[5] Ooka Y , Kanai F , Okabe S *et al* Gadoxetic acid‐enhanced MRI compared with CT during angiography in the diagnosis of hepatocellular carcinoma. Magn. Reson. Imaging. 2013; 31: 748–54. 23218794 10.1016/j.mri.2012.10.028

[6] Huppertz A , Haraida S , Kraus A *et al* Enhancement of focal liver lesions at gadoxetic acid‐enhanced MR imaging: correlation with histopathologic findings and spiral CT‐initial observations. Radiology. 2005; 234: 468–78. 15591431 10.1148/radiol.2342040278

[7] Asayama Y , Tajima T , Nishie A *et al* Uptake of Gd‐EOB‐DTPA by hepatocellular carcinoma: radiologic‐pathologic correlation with special reference to bile production. Eur. J. Radiol. 2011; 80: e243–8. 21109378 10.1016/j.ejrad.2010.10.032

[8] Narita M , Hatano E , Arizono S *et al* Expression of OATP1B3 determines uptake of Gd‐EOB‐DTPA in hepatocellular carcinoma. J. Gastroenterol. 2009; 44: 793–8. 19404564 10.1007/s00535-009-0056-4

[9] Miura T , Ban D , Tanaka S *et al* Distinct clinicopathological phenotype of hepatocellular carcinoma with ethoxybenzyl‐magnetic resonance imaging hyperintensity: association with gene expression signature. Am. J. Surg. 2015; 210: 561–9. 26105803 10.1016/j.amjsurg.2015.03.027

[10] Kitao A , Matsui O , Yoneda N *et al* Gadoxetic acid‐enhanced magnetic resonance imaging reflects co‐activation of β‐catenin and hepatocyte nuclear factor 4α in hepatocellular carcinoma. Hepatol. Res. 2018; 48: 205–16. 28488786 10.1111/hepr.12911

[11] Chou YC , Lao IH , Hsieh PL *et al* Gadoxetic acid‐enhanced magnetic resonance imaging can predict the pathologic stage of solitary hepatocellular carcinoma. World J. Gastroenterol. 2019; 25: 2636–49. 31210715 10.3748/wjg.v25.i21.2636 PMC6558433

[12] Kamiyama T , Nakanishi K , Yokoo H *et al* Perioperative management of hepatic resection toward zero mortality and morbidity: analysis of 793 consecutive cases in a single institution. J. Am. Coll. Surg. 2010; 211: 443–9. 20822741 10.1016/j.jamcollsurg.2010.06.005

[13] Kitao A , Zen Y , Matsui O *et al* Hepatocellular carcinoma: signal intensity at gadoxetic acid‐enhanced MR imaging ‐ correlation with molecular transporters and histopathologic features. Radiology. 2010; 256: 817–26. 20663969 10.1148/radiol.10092214

[14] Yamashita T , Kitao A , Matsui O *et al* Gd‐EOB‐DTPA‐enhanced magnetic resonance imaging and alpha‐fetoprotein predict prognosis of early‐stage hepatocellular carcinoma. Hepatology. 2014; 60: 1674–85. 24700365 10.1002/hep.27093 PMC4142120

[15] Ariizumi S , Ban D , Abe Y *et al* High‐signal‐intensity MR image in the hepatobiliary phase predicts long‐term survival in patients with hepatocellular carcinoma. Anticancer Res. 2019; 39: 4219–25. 31366509 10.21873/anticanres.13583

[16] Vander BS , Libbrecht L , Blokzijl H *et al* Diagnostic and pathogenetic implications of the expression of hepatic transporters in focal lesions occurring in normal liver. J. Pathol. 2015; 207: 471–82. 10.1002/path.1852 16161006

[17] Hatziapostolou M , Polytarchou C , Aggelidou E *et al* An HNF4α‐miRNA inflammatory feedback circuit regulates hepatocellular oncogenesis. Cell. 2011; 147: 1233–47. 22153071 10.1016/j.cell.2011.10.043 PMC3251960

[18] Chen Y , Qin X , Long L *et al* Diagnostic value of Gd‐EOB‐DTPA‐enhanced MRI for the expression of Ki67 and microvascular density in hepatocellular carcinoma. J. Magn. Reson. Imaging. 2020; 51: 1755–63. 31675163 10.1002/jmri.26974

[19] Di Sandro S , Sposito C , Lauterio A *et al* Proposal of prognostic survival models before and after liver resection for hepatocellular carcinoma in potentially transplantable patients. J. Am. Coll. Surg. 2018; 226: 1147–59. 29574178 10.1016/j.jamcollsurg.2018.03.025

[20] Unal E , Akata D , Karcaaltincaba M . Liver function assessment by magnetic resonance imaging. Semin. Ultrasound CT MR. 2016; 37: 549–60. 27986173 10.1053/j.sult.2016.08.006

[21] Kim KA , Kim MJ , Jeon HM *et al* Prediction of microvascular invasion of hepatocellular carcinoma: usefulness of peritumoral hypointensity seen on gadoxetate disodium‐enhanced hepatobiliary phase images. J. Magn. Reson. Imaging. 2012; 35: 629–34. 22069244 10.1002/jmri.22876

[22] Feng ST , Jia Y , Liao B *et al* Preoperative prediction of microvascular invasion in hepatocellular cancer: a radiomics model using Gd‐EOB‐DTPA‐enhanced MRI. Eur. Radiol. 2019; 29: 4648–59. 30689032 10.1007/s00330-018-5935-8

[23] Huang M , Liao B , Xu P *et al* Prediction of microvascular invasion in hepatocellular carcinoma: preoperative Gd‐EOB‐DTPA‐dynamic enhanced MRI and histopathological correlation. Contrast Media Mol. Imaging. 2018: 9674565 10.1155/2018/9674565.eCollection2018. 29606926 PMC5828041

[24] Ahn SJ , Kim JH , Park SJ , Kim ST , Han JK . Hepatocellular carcinoma: preoperative gadoxetic acid–enhanced MR imaging can predict early recurrence after curative resection using image features and texture analysis. Abdom. Radiol. 2019; 44: 539–48. 10.1007/s00261-018-1768-9 30229421

[25] Zhang L , Yu X , Wei W *et al* Prediction of HCC microvascular invasion with gadobenate‐enhanced MRI: correlation with pathology. Eur. Radiol. 2020; 30: 5327–36. 32367417 10.1007/s00330-020-06895-6

[26] Shin SK , Kim YS , Shim YS *et al* Peritumoral decreased uptake area of gadoxetic acid enhanced magnetic resonance imaging and tumor recurrence after surgical resection in hepatocellular carcinoma: a STROBE‐compliant article. Medicine (Baltimore). 2017; 96: e7761. 28816953 10.1097/MD.0000000000007761 PMC5571690

[27] Shimada S , Kamiyama T , Yokoo H *et al* Clinicopathological characteristics of hepatocellular carcinoma with microscopic portal venous invasion and the role of anatomical liver resection in these cases. World J. Surg. 2017; 41: 2087–94. 28271260 10.1007/s00268-017-3964-0

