# Supplementary material for: Impact of gadolinium‐ethoxybenzyl‐diethylenetriamine pentaacetic acid‐enhanced magnetic resonance imaging on the prognosis of hepatocellular carcinoma after surgery
Source: JGH Open. 2020 Oct 30;5(1):41–9. doi: 10.1002/jgh3.12444 (PMC7812518; doi:10.1002/jgh3.12444)
Supplement: Supplementary file 1 — Table S1. Clinicopathological characteristics of patients in the high RHBPP and low RHBPP groups. [file JGH3-5-41-s001.docx]

**Supplementary table 1. Clinicopathological characteristics of patients in the high RHBPP and low RHBPP groups**

| Characteristic | High RHBPP  (n = 60) | | Low RHBPP  (n = 157) | *p* |
| --- | --- | --- | --- | --- |
| **Epidemiology**  Age < 60  ≥ 60  Sex Male | | 11 (18%)  49 (82%)  49 (82%) | 24 (15%)  133 (85%)  128 (82%) | *0.58*  *0.98* |
| Female | | 11 (18%) | 29 (18%) |  |
| HBs-Ag Positive | | 17 (28%) | 42 (27%) | *0.81* |
| Negative | | 43 (72%) | 115 (73%) |  |
| HCV-Ab Positive | | 16 (27%) | 38 (24%) | *0.70* |
| Negative | | 44 (73%) | 119 (76%) |  |
| NBNC 　 Yes | | 27 (45%) | 79 (50%) | *0.48* |
| No | | 33 (55%) | 78 (50%) |  |
| **Biochemical Factors** | |  |  |  |
| Platelets <80,000/mm³ | | 2 (3%) | 10 (6%) | *0.38* |
| ≥ 80,000/mm³ | | 58 (97%) | 147 (94%) |  |
| Albumin　 < 3.5 g/dl | | 7 (12%) | 9 (6%) | *0.13* |
| ≥ 3.5 g/dl | | 53 (88%) | 148 (94%) |  |
| Total bilirubin ≥ 1.0 mg/dl | | 12 (20%) | 30 (19%) | *0.88* |
| < 1.0 mg/dl | | 48 (80%) | 127 (81%) |  |
| PT　　< 80% | | 8 (13%) | 13 (8%) | *0.26* |
| ≥ 80% | | 52 (87%) | 144 (92%) |  |
| ChE　　< 250 IU/l | | 20 (33%) | 58 (37%) | *0.62* |
| ≥ 250 IU/l | | 40 (67%) | 99 (63%) |  |
| ICGR15 ≥ 15% | | 26 (43%) | 68 (43%) | *0.99* |
| < 15% | | 34 (57%) | 89 (57%) |  |
| AFP　　≥ 20 ng/ml | | 20 (33%) | 51 (32%) | *0.90* |
| < 20 ng/ml | | 40 (67%) | 106 (68%) |  |
| PIVKA-II ≥100 mAU/ml | | 32 (53%) | 77 (49%) | *0.57* |
| < 100 mAU/ml | | 28 (47%) | 80 (51%) |  |
| **Tumor Factors** | |  |  |  |
| Tumor size ≥ 5 cm | | 16 (27%) | 59 (38%) | *0.13* |
| < 5 cm | | 44 (73%) | 98 (62%) |  |
| Macroscopic type Simple nodular | | 29 (48%) | 80 (51%) | *0.72* |
| Others  **Histological Factors** | | 31 (52%) | 77 (49%) |  |
| Growth type Expansive growth | | 58 (97%) | 153 (97%) | *0.75* |
| Invasive growth | | 2 (3%) | 4 (3%) |  |
| Tumor capsule fc(+) | | 40 (67%) | 112 (71%) | *0.50* |
| fc(-) | | 20 (3%) | 45 (29%) |  |
| Separation form sf(+) | | 39 (65%) | 104 (66%) | *0.86* |
| sf(-) | | 21 (35%) | 53 (34%) |  |
| Serosa infiltration s0 | | 51 (85%) | 148 (94%) | *0.02* |
| s1-3 | | 9 (15%) | 9 (6%) |  |
| Differentiation Poor | | 29 (48%) | 48 (31%) | *0.01* |
| Others | | 31 (52%) | 109 (69%) |  |
| PVI Yes  No  HVI Yes  No  Fibrosis f3/4  　　　　　　　　　f0-2 | | 27 (45%)  33 (55%)  10 (17%)  50 (83%)  24 (40%)  36 (60%) | 15 (10%)  142 (90%)  9 (6%)  148 (94%)  54 (34%)  103 (66%) | *<0.01*  *0.01*  *0.44* |

RHBPP, ratio of the maximum tumor diameter including peritumoral hypointensity (the portion showing hypointensity around the tumor) measured on HBP images to the maximum tumor diameter measured on precontrast T1-weighted images; HBs-Ag, HBs-antigen; HCV-Ab, HCV antibody; NBNC, patients without HBV and HCV; PT, prothrombin time; ChE, cholinesterase; ICGR15, indocyanine green retention rate at 15 min; AFP, alpha fetoprotein; PIVKA-II, protein induced by vitamin K absence-II; PVI, portal venous invasion; HVI, hepatic venous invasion; f3, bridging fibrosis; f4, cirrhosis.
