# Supplementary material for: Impact of gadolinium‐ethoxybenzyl‐diethylenetriamine pentaacetic acid‐enhanced magnetic resonance imaging on the prognosis of hepatocellular carcinoma after surgery
Source: JGH Open. 2020 Oct 30;5(1):41–9. doi: 10.1002/jgh3.12444 (PMC7812518; doi:10.1002/jgh3.12444)
Supplement: Supplementary file 2 — Table S2. Prognostic factors for survival and recurrence. [file JGH3-5-41-s002.docx]

**Supplementary table 2. Prognostic factors for survival and recurrence**

| Characteristic | Overall survival | | | Relapse-free survival | | | |
| --- | --- | --- | --- | --- | --- | --- | --- |
|  | Univariate (*p*) | | Multivariate (*p*) (hazard ratio)  (95% CI) | | Univariate　(*p*) | Multivariate (*p*)  (hazard ratio)  (95% CI) | |
| **Epidemiology** | |  |  | |  | |  |
| Age　≥ 60 | | *0.25* |  | | *0.82* | |  |
|  | |  |  | |  | |  |
| Sex Male | | *0.41* |  | | *0.39* | |  |
| HBs-Ag Positive  HCV-Ab Positive  NBNC | | *0.28*  *0.85*  *0.20* |  | | *0.50*  *0.47*  *0.22* | |  |
| **Biochemical Factors** | |  |  | |  | |  |
| Platelets < 80,000/mm³ | | *0.52* |  | | *0.42* | |  |
|  | |  |  | |  | |  |
| Albumin < 3.5 g/dl | | *<0.01* | *0.08* | | *0.01* | | *0.68* |
|  | |  | (2.131) | |  | | (1.164) |
|  | |  | (0.906-5.011) | |  | | (0.553-2.451) |
| Total bilirubin ≥ 1.0 mg/dl  PT < 80% | | *0.19*  *<0.01* | *0.37* | | *0.88*  *0.07* | |  |
|  | |  | (1.501) | |  | |  |
|  | |  | (0.616-3.659) | |  | |  |
| ChE < 250 IU/l | | *<0.01* | *<0.01* | | *<0.01* | | *<0.01* |
|  | |  | (3.953) | |  | | (2.754) |
|  | |  | (1.781-8.774) | |  | | (1.750-4.332) |
| ICGR15 ≥ 15% | | *0.03* | *0.74* | | *0.02* | | *0.27* |
|  | |  | (0.894)  (0.460-1.740) | |  | | (1.262)  (0.830-1.919) |
| AFP ≥ 20 ng/ml | | *0.25* |  | | *0.03* | | *0.07* |
|  | |  |  | |  | | (1.488) |
|  | |  |  | |  | | (0.965-2.294) |
| PIVKA-II ≥100 mAU/ml | | *<0.01* | *0.01* | | *<0.01* | | *0.02* |
|  | |  | (2.309) | |  | | (1.708) |
|  | |  | (1.149-4.637) | |  | | (1.073-2.718) |
| **EOB-MRI**  RHBPP ≥1.036  DI  **Tumor Factors** | | *0.01*  *0.71* | *<0.01*  (2.724)  (1.291-5.749) | | *<0.01*  *0.56* | | *<0.01*  (2.976)  (1.896-4.670) |
| Tumor size ≥ 5 cm | | *0.10* |  | | *<0.01* | | *0.03* |
|  | |  |  | |  | | (1.651) |
|  | |  |  | |  | | (1.042-2.616) |
| Macroscopic type | | *0.10* |  | | *<0.01* | | *0.25* |
| Others | |  |  | |  | | (1.295) |
|  | |  |  | |  | | (0.832-2.015) |

HBs-Ag, HBs-antigen; HCV-Ab, HCV antibody; NBNC, patients without HBV and HCV; PT, prothrombin time; ChE, cholinesterase; ICGR15, indocyanine green retention rate at 15 min; AFP, alpha-fetoprotein; PIVKA-II, protein induced by vitamin K absence-II; RHBPP, ratio of the maximum tumor diameter including peritumoral hypointensity (the portion showing hypointensity around the tumor) measured on HBP images to the maximum tumor diameter measured on precontrast T1-weighted images; DI, decreased tumor intensity compared with liver intensity.
